# Structure and evolution of the 4-helix bundle domain of Zuotin, a J-domain protein co-chaperone of Hsp70

**DOI:** 10.1371/journal.pone.0217098

**Published:** 2019-05-15

**Authors:** Om Kumar Shrestha, Ruchika Sharma, Bartlomiej Tomiczek, Woonghee Lee, Marco Tonelli, Gabriel Cornilescu, Milena Stolarska, Lukasz Nierzwicki, Jacek Czub, John L. Markley, Jaroslaw Marszalek, Szymon J. Ciesielski, Elizabeth A. Craig

**Affiliations:** 1 Department of Biochemistry, University of Wisconsin-Madison, Madison, Wisconsin, United States of America; 2 Laboratory of Evolutionary Biochemistry, Intercollegiate Faculty of Biotechnology, University of Gdansk and Medical University of Gdansk, Gdansk, Poland; 3 National Magnetic Resonance Facility at Madison, University of Wisconsin-Madison, Madison, Wisconsin, United States of America; 4 Department of Physical Chemistry, Gdansk University of Technology, Gdansk, Poland; Indian Institute of Science, INDIA

## Abstract

The J-domain protein Zuotin is a multi-domain eukaryotic Hsp70 co-chaperone. Though it is primarily ribosome-associated, positioned at the exit of the 60S subunit tunnel where it promotes folding of nascent polypeptide chains, Zuotin also has off-ribosome functions. Domains of Zuotin needed for 60S association and interaction with Hsp70 are conserved in eukaryotes. However, whether the 4-helix bundle (4HB) domain is conserved remains an open question. We undertook evolutionary and structural approaches to clarify this issue. We found that the 4HB segment of human Zuotin also forms a bundle of 4 helices. The positive charge of Helix I, which in *Saccharomyces cerevisiae* is responsible for interaction with the 40S subunit, is particularly conserved. However, the C-termini of fungal and human 4HBs are not similar. In fungi the C-terminal segment forms a plug that folds back into the bundle; in *S*. *cerevisiae* it plays an important role in bundle stability and, off the ribosome, in transcriptional activation. In human, C-terminal helix IV of the 4HB is extended, protruding from the bundle. This extension serves as a linker to the regulatory SANT domains, which are present in animals, plants and protists, but not fungi. Further analysis of Zuotin sequences revealed that the plug likely arose as a result of genomic rearrangement upon SANT domain loss early in the fungal lineage. In the lineage leading to *S*. *cerevisiae*, the 4HB was subjected to positive selection with the plug becoming increasingly hydrophobic. Eventually, these hydrophobic plug residues were coopted for a novel regulatory function—activation of a recently emerged transcription factor, Pdr1. Our data suggests that Zuotin evolved off-ribosome functions twice—once involving SANT domains, then later in fungi, after SANT domain loss, by coopting the hydrophobic plug. Zuotin serves as an example of complex intertwining of molecular chaperone function and cell regulation.

## Introduction

J-domain proteins (JDPs) are obligate co-chaperones of Hsp70-based molecular chaperone systems [1, 2]. Such chaperone machineries are ubiquitous, functioning in diverse biological processes [3–5]. Zuotin is the JDP of the eukaryotic ribosome-associated Hsp70 system, which facilitates folding of nascent polypeptide chains [6, 7]. Zuotin binds near the exit site of the ribosome tunnel of the 60S subunit from which nascent chains emerge [8]. Like other JDP/Hsp70 systems, Zuotin’s J-domain is required for stimulation of the ATPase activity of its partner Hsp70, which facilitates efficient Hsp70 interaction with its substrate proteins [9, 10]. Zuotin is called Zuo1 in fungi and ZRF1 (Zuotin-related factor), Mpp11 or DNAJC2 in animals. In animals, plants and protists, but not fungi, Zuotin orthologs have a C-terminal, ~200 residue, segment containing SANT domains [11, 12]—domains typically involved in protein-protein interactions [13]. An early phylogenetic analysis of Zuotin established that fungal Zuotin evolved from a progenitor that had SANT domains, strongly suggesting that these domains were lost during fungal evolution [11]. Changes that occurred in Zuotin upon SANT domain loss is one focus of this report.

**Fig 1:**
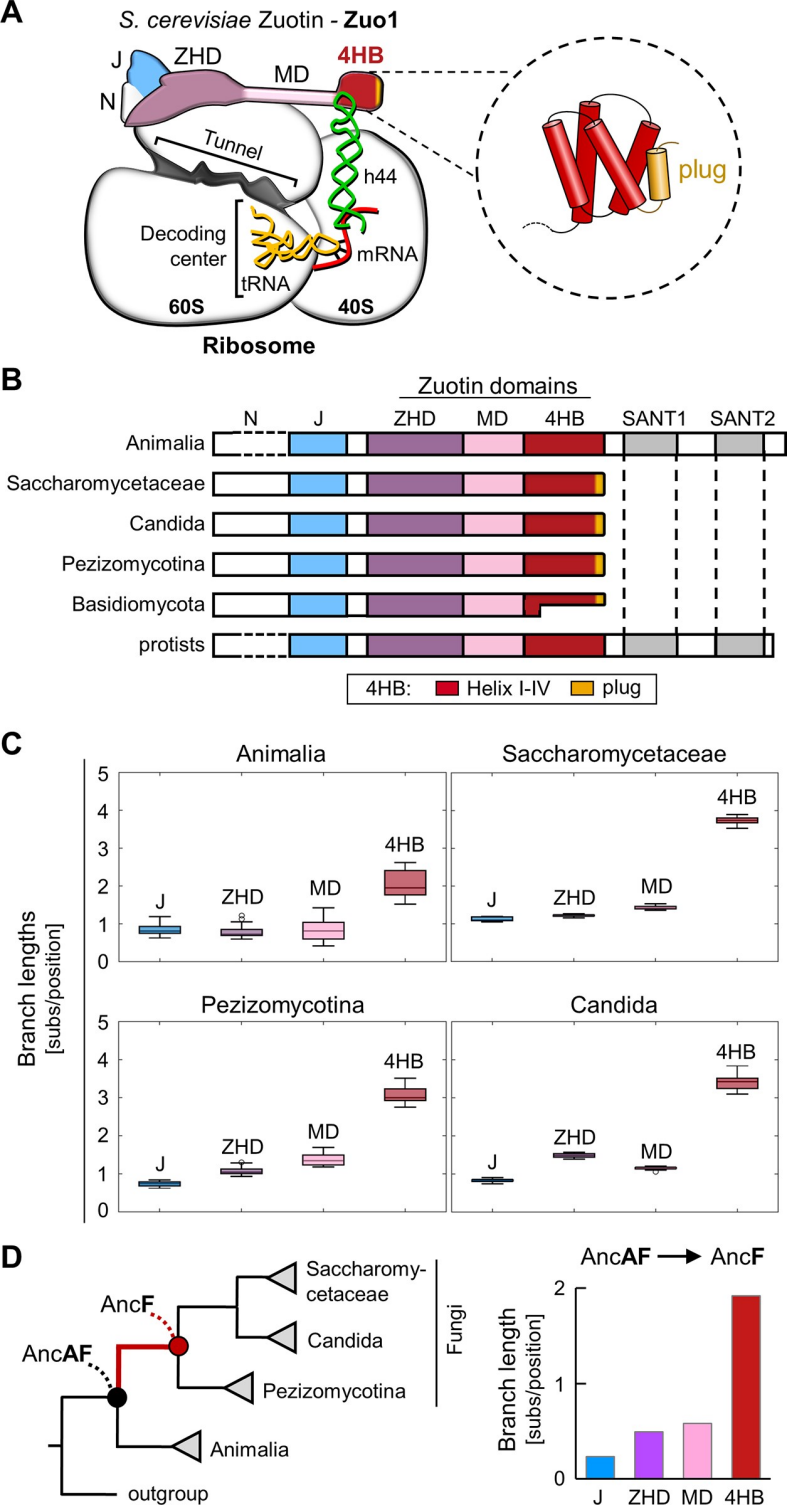
Conservation of Zuotin domains. (A) Diagram of association of Zuotin from *Saccharomyces cerevisiae* (Zuo1) with the ribosome. Zuo1 interacts with both subunits. Site of interaction with the 60S subunit via the Zuotin homology domain (ZHD, purple) is very close to the exit of the tunnel through which the nascent polypeptide traverses. The N terminus (N, white), which interacts with the atypical Hsp70 Ssz1 (not shown), is adjacent to the J domain (J, blue). The ZHD is adjacent to a long α-helix constituting the middle domain (MD, pink), which connects the ZHD and the C-terminal four-helix bundle (4HB, red). Regions of Zuo1 at the junction of the MD and Helix I of the 4HB interact with the 40S subunit, at the end of ribosomal RNA helix 44 (h44), in green, which emanates from the decoding center. Structure of *S*. *cerevisiae* 4HB shown at right (PDB ID: 2LWX); hydrophobic plug in gold. (B) Comparison of domains of Zuotin homologs from 104 eukaryotic species: SANT domains (SANT1 and SANT2); other domains are labeled as in A. Dotted lines indicate length variability of N domain. A subset of Basidiomycota species has a truncated 4HB region, as indicated by the different lengths of the 4HB segment. (C) Evolutionary rates, estimated based on the branch lengths of domain specific phylogenies (substitutions/position (subs/pos)), for individual domains of Zuotin homologs from indicated clades (S3 Fig) are represented as a box-and-whisker plot: 25th and 75th percentiles (box), within 1.5 of the interquartile range (whisker); outliners (white circles). In all cases the evolutionary rate of the 4HB domain is significantly higher (p < 0.01, Mann–Whitney U test) than that of each of the other domains. (D) (left) Phylogenetic tree illustrating relatedness among monophyletic groups of species used in our analyses. Nodes representing common ancestors of Animalia and Fungi (AncAF) and Fungi (AncF) are indicated by black and red dots, respectively. (right) Lengths of branches connecting AncAF with AncF (shown in red at left) from domain specific trees presented in Supplementary data (S3 Fig).

This ribosome-associated chaperone system has been most thoroughly studied in the budding yeast *Saccharomyces cerevisiae*. In addition to interacting with the 60S subunit through its Zuotin Homology Domain (ZHD), Zuo1 binds the 40S, via the intervening middle domain (MD) and Helix I of its C-terminal 4-helix bundle (4HB) [14–17] (Fig 1A). These 40S-binding MD and 4HB segments interact with the tip of ribosomal RNA helix 44, which emanates from the decoding center, the site where incoming tRNA anticodons are matched with mRNA codons. Consistent with this positioning, absence of the Zuo1-40S interaction results in changes in the accuracy of translation–increased readthrough of stop codons and decreased frameshifting [16].

Though predominantly found associated with cytosolic ribosomes, Zuotin has been shown to have functions off the ribosome in the nuclei of human and budding yeast cells [8, 18]. However, different Zuotin sequences are implicated in these activities. Consistent with known SANT domain involvement in chromatin remodeling [8, 19, 20], Zuotin is implicated in functions ranging from regulation of cell differentiation, development and tumorigenesis in protists and animals [21–23]. In *S*. *cerevisiae* Zuotin (Zuo1), which lacks SANT domains, activates the transcription factor Pdr1 [24], which is constitutively bound to DNA and has an activation domain that binds signaling molecules [25]. The very C-terminal 13 residues of the 4HB domain of Zuo1 are sufficient for Pdr1 activation [26]. This hydrophobic 13-residue segment folds back and binds like a 'plug' in a hydrophobic groove between Helices II and IV, stabilizing the 4HB structure [15, 16]. Each of the 7 hydrophobic residues of the plug is important for activation of Pdr1 by Zuo1 [26]. This plug-dependent activation of Pdr1 is a recently evolved function of Zuo1, as Pdr1, a new member of a large family of fungal transcription factors, is only present in a subset of species closely related to *S*. *cerevisiae* [27]. In part this report focuses on the evolutionary history of the 4HB plug and its novel function in transcriptional activation.

We took evolutionary and structural approaches to better understand similarities and differences between fungal and animalian Zuotin 4HB regions. We determined that the 4HB region of human Zuotin, which lies between the MD and SANT domains, forms a 4-helix bundle. However, while most of the 4HB sequence is conserved throughout evolution, the very C-terminal region is not. Unlike the plug found in fungi at this position, this segment protrudes from the human Zuotin bundle connecting it with the SANT domains. Our evolutionary analysis also indicates that the hydrophobic plug evolved early in the fungi lineage, upon loss of the SANT domains. Later, in the *S*. *cerevisiae* lineage, it became particularly hydrophobic—with these hydrophobic residues being required for Zuo1’s ability to activate the Pdr1 transcription factor.

## Results

### 4HB domain evolved faster than other Zuotin domains

To better understand the evolution of the 4HB domain, we first collected Zuotin amino acid sequences from a wide range of organisms—animals, fungi and protists (S1 Fig). Our fungal dataset contained 4 subgroups: Saccharomycetaceae, Candida and Pezizimycotina, which belong to the phylum Ascomycota and contain the model organisms, *S*. *cerevisiae*, *Candida albicans* and *Chaetomium thermophilum*, respectively, as well as Basidiomycota, which includes many mushroom species. We aligned the Zuotin sequences in our dataset. The J-domain, ZHD and MD domains aligned well across species. However, differences were apparent in other segments. First, consistent with previously published data [11, 12], SANT domains are present in Zuotin from animal and protists, but not fungal, species (Fig 1B). Second, the segment N-terminal to the J-domain was found to be variable in length, and was not considered further. Third, although most of the fungal 4HB segment align with animal and protist sequences, two disparities were noted: (i) the plug sequence does not align with the rest of the 4HB; (ii) in a monophyletic subgroup of Basidiomycota species, the 4HB region is truncated, with only 9–16 residues present (Fig 1B, S1 Fig).

To analyze the rates of sequence evolution of Zuotin domains we carried out phylogenetic analyses. First, we used our aligned sequences to reconstruct protein phylogeny for full-length Zuotin, using the protists sequences as an outgroup. Overall, the branching order of the resulting tree is consistent with accepted organismal phylogeny (S2 Fig). Next, we reconstructed individual phylogenetic trees for the J, ZHD, MD and 4HB domains, omitting Basidiomycota sequences because of the 4HB truncation (S3 Fig). The branches on the 4HB tree are longer than those of the other domain trees. As longer branch lengths are indicative of greater numbers of amino acid substitutions, they suggest a higher rate of sequence evolution. To more rigorously assess evolution rates, we calculated branch lengths for each species in each domain tree. For each group of species—Animalia, Pezizomycotina, Candida and Saccharomycetaceae—rates are significantly higher (p <0.01) for the 4HB domains than those for other domains (Fig 1C). The differences were most evident in fungi, with the highest rates in Saccharomycetaceae and Candida.

When comparing domain trees, we also noted that the earliest branch—the one leading from the ancestor of animals and fungi (AncAF) to the common ancestor of the fungi included in our analysis (AncF)—is markedly longer on the 4HB tree than on the other domain trees (Fig 1D, S3 Fig). This greater length indicates that multiple amino acid substitutions occurred within the 4HB region in fungi, soon after separation from animals. Thus, not only did the 4HB evolve faster than the J, ZHD and MD domains, many of the changes occurred in the timeframe of SANT domain loss in the progenitor of fungi.

### 4HB region of human Zuotin forms a 4-helix bundle that lacks a plug

**Fig 2:**
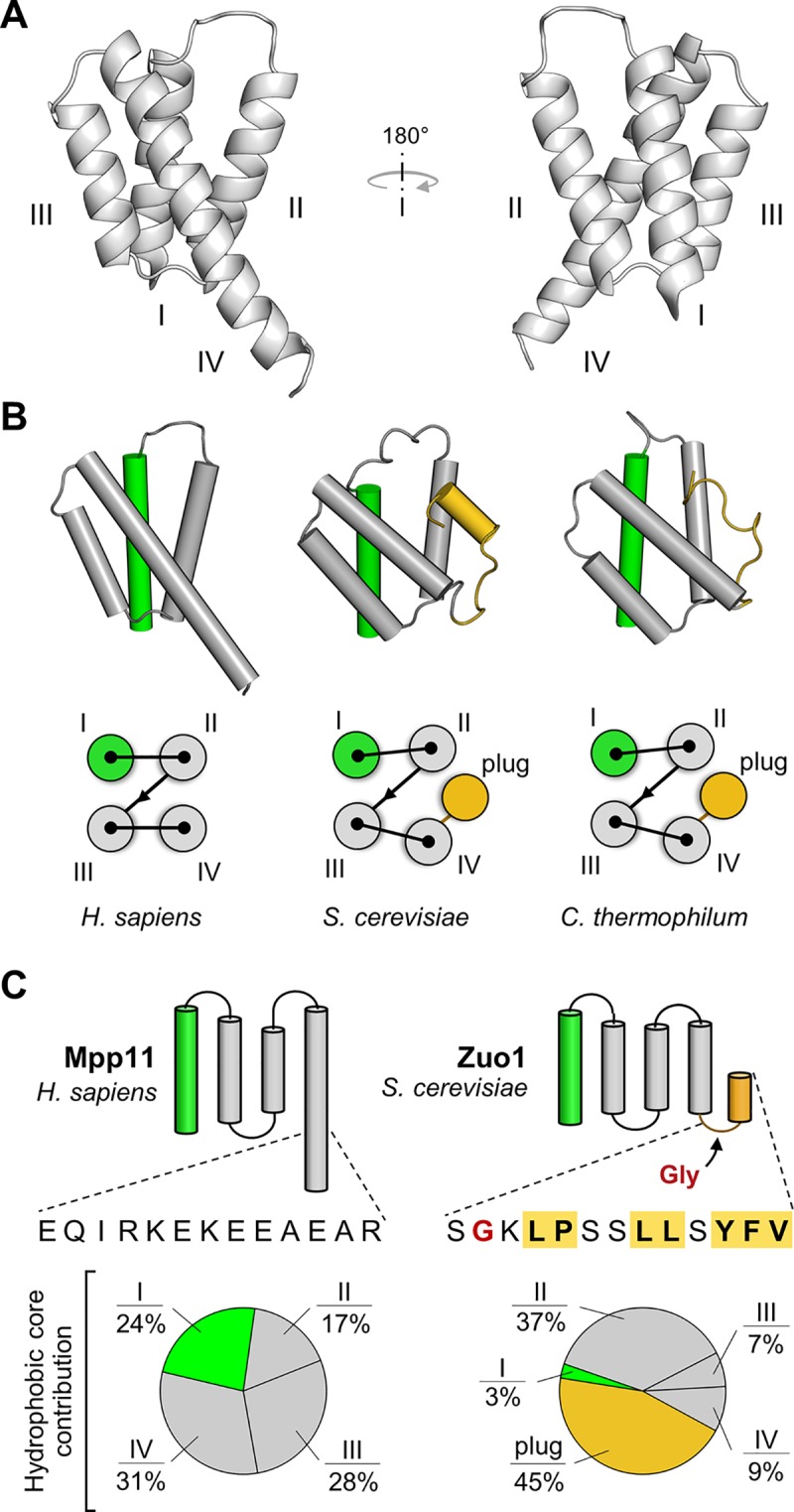
Structure of the 4HB domain. (A) Structure of the 4HB domain of human Zuotin (Mpp11_346-432_) determined by NMR (PDB ID: 6CGH). Four helices (I-IV) form a compact structure with the longest helix, Helix IV, protruding out of the bundle. (B) Comparison of 4HBs of human and fungal Zuotin. (top) 4HBs are presented in the same orientation, with helices represented as cylinders. The C-terminal plug segment following Helix IV in fungal Zuotins are highlighted in gold; Helix I is in green. *S*. *cerevisiae* (PDB ID: 2LWX) and *Chaetomium thermophilum* (PDB ID: 4GMQ). (bottom) Schematic of organization of 4HBs—Helices I-IV and fungal plugs. (C) Contribution to the hydrophobic core. (top) Graphic of organization of Mpp11 and Zuo1 4HB, highlighting the C-termini. The C-terminal 13 residues of the bundles are shown. Residues of the *S*. *cerevisiae* plug shown to be required for Pdr1 activation are highlighted in yellow, with bold lettering. Glycine that allows the plug to fold back into the bundle in red. (bottom) Pie chart presenting individual contribution to the 4HB hydrophobic core by Helices I, II, III and IV, and for Zuo1 the hydrophobic plug, estimated based on their calculated individual hydrophobic surfaces using the NMR-derived ensembles–PDB ID: 6CGH (human Mpp11) and PDB ID: 2LWX (*S*. *cerevisiae* Zuo1).

The rapid evolution of the 4HB domain raises the question of whether the 4HB region of human Zuotin (Mpp11) actually forms a bundle of 4 helices, and, if it does, whether it also contains a C-terminal plug like the fungal orthologs. To resolve this issue, we employed nuclear magnetic resonance (NMR) spectroscopy. We solved the structure of Mpp11_346-432_. This 4HB region fragment, indeed, folds into a 4-helix bundle (Fig 2A, S1 Table). The four helices, connected by short loops, are oriented similarly to those of the two fungal Zuotin 4HBs whose structures have been determined: *S*. *cerevisiae* (PDB ID: 2LWX) [26] and *C*. *thermophilum* (PDB ID: 4GMQ) [14]. The helices are packed lengthwise against each other in a simple up-down-up-down topology, each contributing to the hydrophobic core of the domain (Fig 2B). A notable similarity (Helix I) and a notable difference (extreme C-terminus) are discussed below.

**Fig 3:**
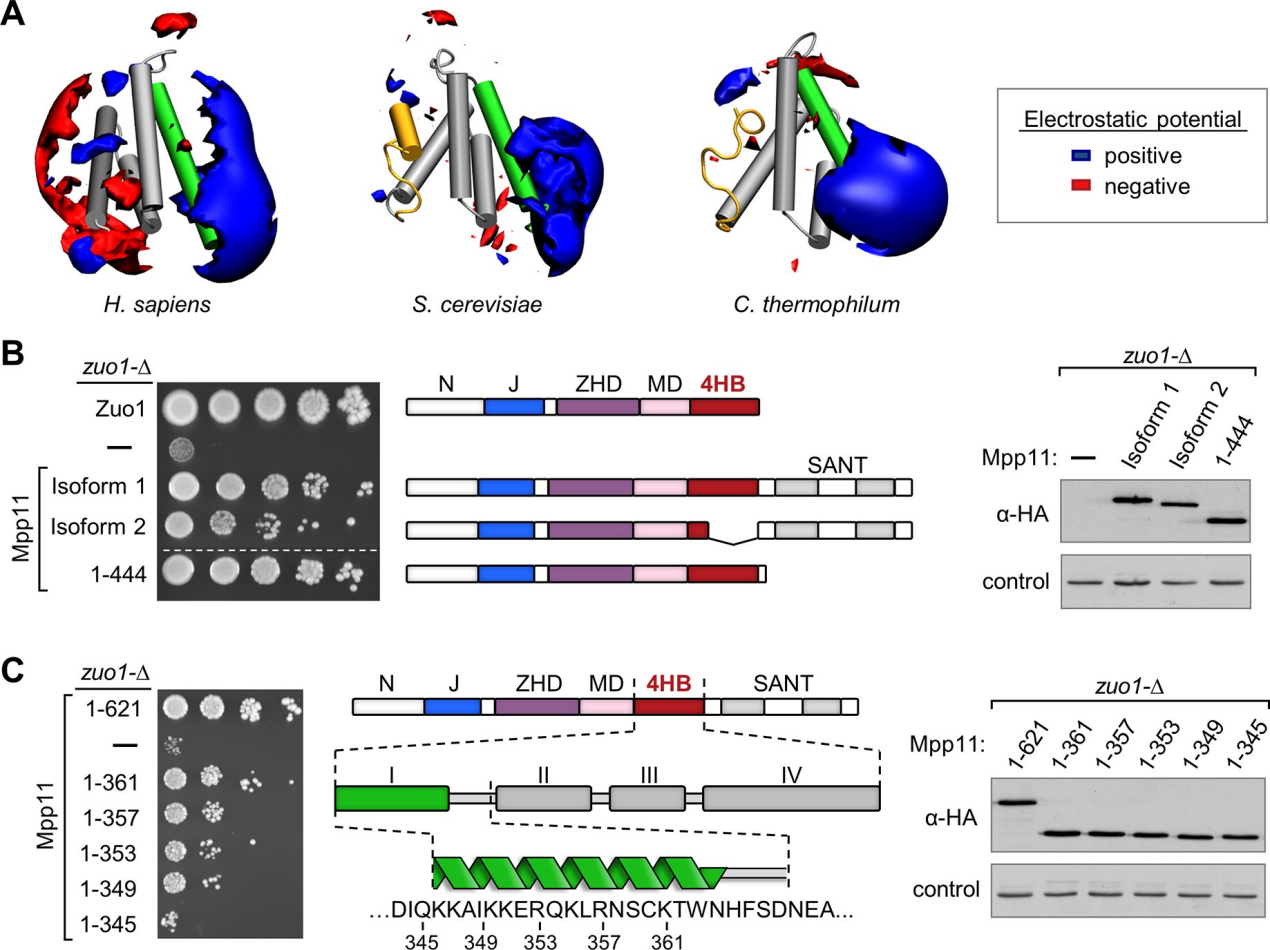
Functional importance of conserved Helix I of the 4HB. (A) Electrostatic isopotential contour maps (blue +2, red -2 kT/e) around the 4HB structures. Helix I in green, hydrophobic plug in the two fungal structures in gold. (B and C) Analysis of the ability of Mpp11 variants to support growth of *S*. *cerevisiae zuo1-Δ* cells. (left) *zuo1-Δ* strains expressing Zou1, HA-tagged Mpp11 or indicated truncated variants were 5-fold serially diluted, spotted on paromomycin-containing plates and incubated at 30°C for 5 (B) or 6 (C) days. (middle) Schematic representation of domain composition of Zou1, Mpp11 and Mpp11 truncation variants. (right) Cell lysates made from indicated strains were subjected to immunoblot analysis using HA-tag specific, or as a control, Ydj1 specific antibodies. Number of residues present in Mpp11 truncation variants are indicated. Two isoforms of Mpp11 identified in humans were tested in C: Isoform 1, “full-length” Mpp11, residues 1–621 and isoform 2 which lacks codons for residues 362–414, which encode Helices II and III, as well as part of Helix IV. Dotted line in B indicates that a strain not relevant to this manuscript was spliced from the image of the plate.

In both fungal and human 4HBs the N-terminal 9 residue segment of Helix I is particularly enriched in positively charged amino acids—6 residues being either lysine or arginine—resulting in a prominent patch of uniformly positive electrostatic potential (Fig 3A). As this sequence in *S*. *cerevisiae* Zuotin is essential for 40S subunit interaction [16], we tested whether Helix I of Mpp11 is important for its ability to overcome a phenotype of *S*. *cerevisiae* cells lacking Zuo1 (i.e. *zuo1-Δ*)—sensitivity to paromomycin, a cationic aminoglycoside inhibitor of protein synthesis [10, 28] (Fig 3B and 3C). A truncation lacking both the SANT domains and the residues of Helices II-IV of the 4HB (Mpp11_1-361_) was nearly as effective as full-length protein (Fig 3C). Cells expressing variant Mpp11_1-345_, which completely lacks Helix I sequences [29], grew as poorly as *zuo1-Δ* cells carrying only an empty plasmid. Isoform 2, a human splicing variant that lacks most Helix II-IV sequences [21], substantially rescues the paromomycin sensitivity of *S*. *cerevisiae zuo1-Δ* (Fig 3B). These results are consistent with the positive charge of Helix I being important for ribosome association of Mpp11, as for *S*. *cerevisiae* Zuo1.

The C-terminal segments of the fungal and human bundles obviously differ. While the lengths of the first three helices are similar, Mpp11 Helix IV is longer, by more than 50%. Mpp11 Helix IV extends beyond the bundle—in contrast to the fungal structures in which a bend occurs at the glycine at the end of the shorter Helix IV, allowing plug residues to fold back into the core of the bundle (Fig 2B and 2C). Consistent with this, sequence-based secondary structure predictions of the animal sequences in our data set predict a high helical propensity of the Helix IV extension (S4 Fig). This extension is rich in charged, rather than hydrophobic, residues, resulting in a patch of negative electrostatic potential surrounds Helix IV of the Mpp11 4HB that is not present in either fungal structure (Fig 3A and S4 Fig). The function of this charged region is unclear, but may have a structural role via electrostatic interactions with the adjacent SANT domain segment that is absent in fungal Zuotins.

Because of the difference between the C-termini of human and *S*. *cerevisiae* 4HBs, we compared the relative contribution of helices/plug to the structural integrity of the two bundles. Our results indicate that the contribution of individual structural segments to the two bundles is different (Fig 2C). Each of the four helices of human 4HB is a significant contributor, ranging from 28% for Helix III to 17% for Helix II. In contrast, Helices I, III and IV of the *S*. *cerevisiae* bundle, combined, provide only 19% of the buried hydrophobic surface. However, the plug contributes 45%, suggesting that the hydrophobic plug, which is not present in human Zuotin, contributes substantially to the structural stability of the *S*. *cerevisiae* bundle. This idea is consistent with the previous experimental finding that deletion of the three most C-terminal residues of the plug, the hydrophobic amino acids tyrosine, phenylalanine and valine, severely destabilizes the 4HB structure [26].

### 4HB plug competent for Pdr1 activation evolved before emergence of Pdr1

**Fig 4:**
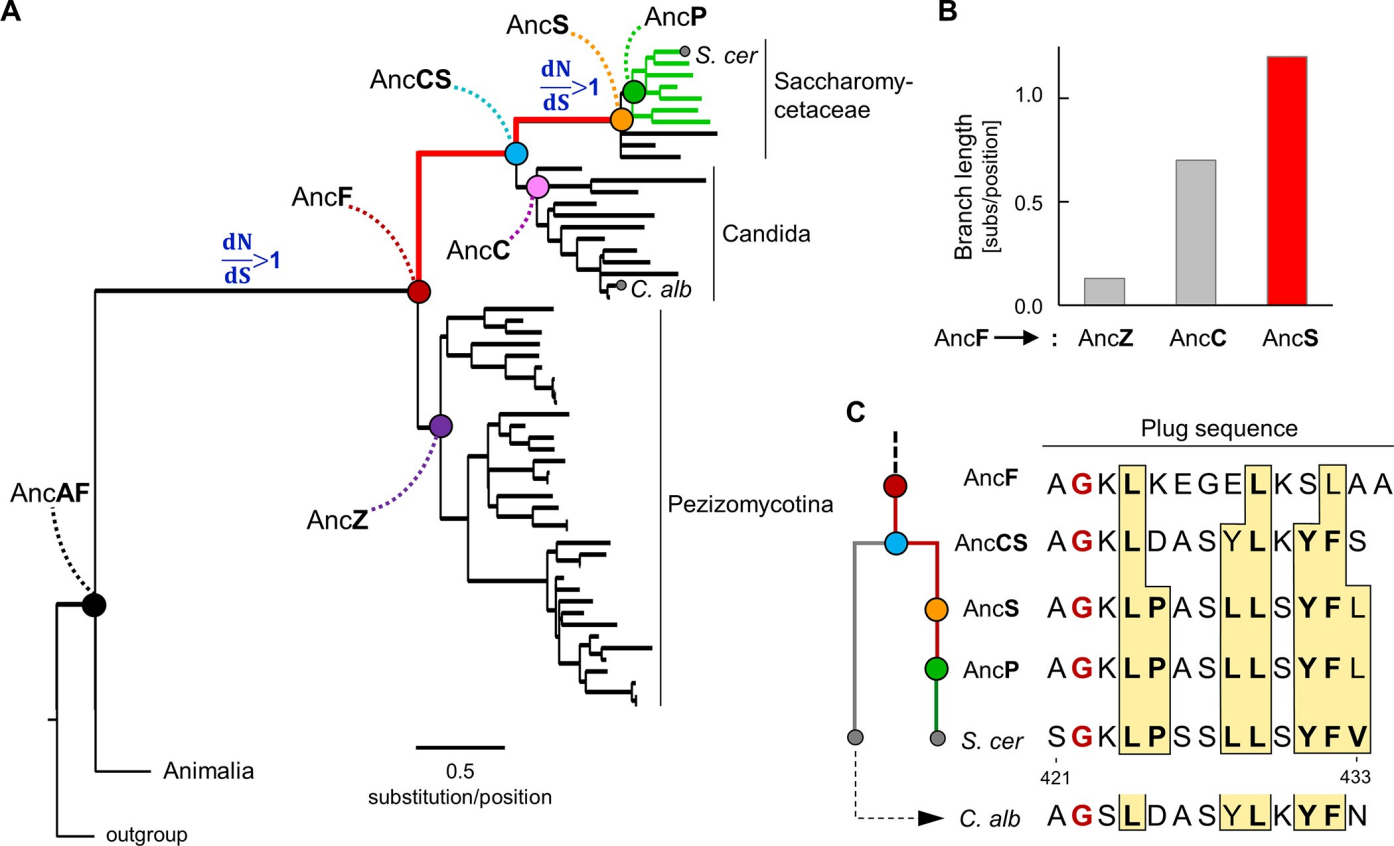
Evolution of 4HB plug. (A) Phylogeny of 4HB domains from Pezizomycotina, Candida and Saccharomycetaceae, with protists used as an outgroup. Nodes representing common ancestors are indicated by dots: Black—Animalia and Fungi (AncAF); red—Fungi (AncF); purple—Pezizomycotina (AncZ); cyan—Candida and Saccharomycetaceae clades (AncCS); pink—Candida (AncC); orange—Saccharomycetaceae (AncS); green—Saccharomycetaceae that harbor Pdr1 transcription factor (AncP). A dN/dS ratio significantly higher than 1 (dN/dS>1) for a given branch of the tree is indicated. All branch specific dN/dS values are presented in Supplementary data (S5 Fig and S6 Fig). Order of the branches representing species is the same as in Supplementary data (S1 Fig). Green branches represent species harboring Pdr1. Branch from AncF to AncS in red. Branches representing *S*. *cerevisiae* (S. cer) and *Candida albicans* (C. alb) are marked by grey dots. (B) Lengths of branches connecting the node representing the common ancestor of Fungi (AncF) with nodes representing the common ancestors of Pezizomycotina (AncZ), Candida (AncC) and Saccharomycetaceae (AncS) as in tree in A. (C) Evolution of the sequence of the plug. (Left) Schematic representation of plug sequence evolution from the common ancestor of Fungi (AncF) to *S*. *cerevisiae* (S. cer) and *C*. *albicans* (C. alb). Common ancestors indicated: Candida and Saccharomycetaceae clades (AncCS), Saccharomycetaceae (AncS), Saccharomycetaceae that harbors Pdr1 transcription factor (AncP). (right) The sequences of reconstructed ancestors, *C*. *albicans* and *S*. *cerevisiae* plugs are shown. Large hydrophobic residues are boxed with yellow background. Specific hydrophobic residues that in the *S*. *cerevisiae* plug have been demonstrated experimentally to be important for Pdr1 activation are in bold.

Because of the structural differences in fungal and human 4HBs we analyzed the 4HB domain tree more extensively. We found that, of the branches in the part of the 4HB tree connecting AncF with the ancestors of Pezizomycotina (AncZ), Candida (AncC) and Saccharomycetaceae (AncS), the longest is the one leading to AncS (Fig 4A and 4B). Because *S*. *cerevisiae*, whose plug activates Pdr1, belongs to Saccharomycetaceae, this markedly greater branch length (i.e. high evolution rate) was of interest to us. Therefore, we tested for positive selection. We assessed ratios of nonsynonymous (dN; coding for a different amino acid) to synonymous (dS; silent), that is dN/dS, nucleotide substitution rates, for each branch of the 4HB tree (S5 Fig; S2 and S3 Table).

We found two episodes of positive selection, that is a dN/dS ratio >1, in the lineage leading to AncS (Fig 4A). One is on the branch leading to the common ancestor of fungi (AncF), coincident with loss of the SANT domains and origin of the plug. The second, more recent one, is on the branch leading from the common ancestor of Candida and Saccharomycetaceae (AncCS) to the ancestor of Saccharomycetaceae (AncS) (Fig 4A). The first episode is not surprising; many amino acid substitutions were likely driven by positive selection when the plug became a novel structural element of the bundle upon loss of the SANT domains. The second episode is of interest because it immediately predated the emergence of Pdr1.

To analyze patterns of plug sequence evolution (S4 Fig), we used ancestral sequence reconstruction [30] to infer the amino acid sequences of ancestral plugs from the ancestor of fungi (AncF) to the ancestor of species harboring Pdr1 (AncP). Two features of the ancestral plugs are evident (Fig 4C; S7 Fig). First, a glycine residue at the N-terminus of the plug is conserved, present even in AncF. This conservation is not surprising, because of glycine’s structural role, evident in the two available fungal structures (Fig 2), that allows the plug to fold back and interact at the Helix II-Helix IV interface. Second, the number of large hydrophobic residues in the plug sequence increases gradually in the *S*. *cerevisiae* lineage (Fig 4C). This increase is particularly interesting, because of the importance of the seven hydrophobic residues of the plug in Pdr1 activation in *S*. *cerevisiae* [26]. In AncF, the plug has only three such residues, while in AncS the plug has 7. Six out of seven residues are the same as in *S*. *cerevisiae*. The 7^th^, the most C-terminal residue in the *S*. *cerevisiae* plug (position 433), is L instead of V. This site is variable among species harboring Pdr1, though always occupied by a hydrophobic residue (S4 Table).

Since *C*. *albicans* branched off before the emergence of Pdr1, we compared its and *S*. *cerevisiae* plug sequences to that of their common ancestor AncCS. The plug sequence did not change as much from AncCS to *C*. *albicans* as it did from AncCS to *S*. *cerevisiae*: two changes in *C*. *albicans*; six changes in *S*. *cerevisiae* (Fig 4C). Moreover, in the *S*. *cerevisiae* lineage two additional hydrophobic residues, both critical for Pdr1 activation, emerged between AncCS and AncS—that is on the branch for which we detected a signature of positive selection. Whereas in *C*. *albicans*, these residues are the same as in the ancestral (AncCS) sequence. These observations are consistent with the idea that plug sequence evolution in the *S*. *cerevisiae* lineage was unique and particularly complex. Remarkably, all hydrophobic residues required for the Pdr1 activation by the plug were already present before Pdr1 itself emerged.

## Discussion

New information regarding the relationship between human and fungal Zuotin, and the evolution of Zuo1’s off-ribosome regulatory function in transcriptional activation emerged from the analyses reported here. Notably, the 4HB is conserved in most eukaryotes. However, while Helix I is particularly conserved, the C-terminal end of the 4HB differs substantially between animals and fungi—in the former it is a linker to the SANT domains, while in a subset of fungi related to *S*. *cerevisiae*, a regulator of transcriptional activity.

### Helix I of the 4HB

Unfortunately, aggregation of Mpp11 in yeast cells prevented our rigorous assessment of ribosome association per se. However, the effect of C-terminal truncations on Mpp11’s ability to substitute for the absence of Zuo1 in *S*. *cerevisiae* is consistent with the idea that Helix I’s role in ribosome association is conserved. In Mpp11 removal of Helices II-IV had little effect on rescue. However, progressive removal of Helix I residues had increasingly severe effects. In *S*. *cerevisiae*, Helix I is required for stable ribosome association, but the rest of the 4-helix bundle sequence is not; the Zuo1_1-363_ truncation, which retains Helix I sequences, associates stably with ribosomes [29]. Furthermore, substitution of charged residues on the surface of Helix I causes destabilization of the Zuo1-ribosome interaction [16]. Interestingly, even in the subgroup of Basidiomycota species having a truncation of the 4HB, at least 9 residues of Helix I, the most positively charged segment, remains. Similarly, the human splicing variant lacks much of the 4HB sequence, but retains Helix I sequences [21].

In *S*. *cerevisiae* Helix I of the 4HB is also implicated in translational fidelity. Consistent with its interaction with the tip of rRNA helix 44 of the 40S subunit that originates from the decoding center [16], absence of the Zuo1-40S interaction results in changes in the fidelity of translation [16]. Considering the conservation of Helix I, such an interaction could well play a role in translational fidelity in most eukaryotes. Consistent with this idea, a decrease in Zuotin levels in human cells leads to an increased sensitivity to aminoglycosides and increased readthrough of stop codons [31].

### The plug of the fungal 4HB

Our data suggests a complex evolutionary history of the 4HB domain in the *S*. *cerevisiae* lineage since loss of the SANT domains in the progenitor of fungi. First, the C-terminal plug evolved; second, its hydrophobicity increased; third, the plug was coopted for transcriptional activation upon emergence of Pdr1. The origin of the plug is unclear, as its short and variable sequence precludes rigorous analyses. Possibly, it evolved from the Helix IV extension. Alternatively, it might have originated from an sequence unrelated to Zuotin, the result of complex sequence rearrangements that took place upon loss of the SANT domains. Regardless of the origin of the plug’s sequence, in all likelihood its emergence was a random event. We speculate that the ancestral 4HB domain after the SANT domains loss was stable on its own, as the human 4HB is stable in the absence of both the SANT domains and a plug. However, once the newly emerged plug began contributing significantly to the hydrophobic core and thus to the structural integrity of the 4HB, a "no-return" situation was created. This can explain why an episode of positive selection coincided with the origin of the plug.

Regardless of the scenario behind the emergence of the plug, further evolutionary changes paved the way for its future cooption for Pdr1 activation. Along the *S*. *cerevisiae* lineage, the plug sequence became particularly enriched in hydrophobic residues. At a late stage of this gradual process, we detected a signature of positive selection, but the factor(s) driving the selected changes are unknown. At this ancestral node, all hydrophobic residues needed for Pdr1 activation were already in place [26]. However, the phylogenetic distribution of Pdr1 [27], which is restricted to a monophyletic group of species related to *S*. *cerevisiae*, indicates that Pdr1 emerged later. Thus, the plug sequence competent for transcriptional activation evolved before the factor which it activates, Pdr1.

The presence of the 4HB domain with a plug competent for Pdr1 activation in the ancestor predating the emergence of Pdr1 could be considered an example of molecular preadaptation [32, 33], a structure that evolved for other reasons, but due to its unique features could be coopted (exapted) for a new function [34]. The existence of a "ready to use" binding interface is considered a feature that often predisposes proteins for cooption [35, 36]. The presence of hydrophobic residues in the plug sequence that allowed it to directly interact with the Pdr1 transcription factor fits this condition well. Overall, our results indicate that evolution of the novel regulatory activity of fungal Zuotin was a complex process, which involved chance events, episodes of positive selection, preadaptation and cooption—making this an illustrative case study of how proteins evolve novel activities [37–39].

### Zuotin regulatory functions

In the bigger picture, it is noteworthy that off-ribosome nuclear regulatory functions for Zuotin evolved at least twice, once at the base of the eukaryotic lineage and once in the fungal lineage after loss of the SANT domains. Though the molecular mechanisms are not the same in divergent eukaryotes, it is tempting to speculate that this unusual functional convergence is connected to Zuotin’s strategic placement on the ribosome—interacting with both subunits: on the 40S Helix I of the 4HB with the tip of ribosomal RNA helix 44 that emanates from the decoding center and on the 60S near the nascent chain exit site, with a possible signaling connection more internally in the tunnel [15, 16]. One can envision how this positioning may allow monitoring and subtle regulation of both the rate of peptide bond formation and the folding state of the nascent chain. Such positioning may also lend itself to more global regulation, for example, when translation is regulated in response to environmental factors. It is intriguing that Pdr1 activation by Zuo1 has been linked to quorum sensing and control of growth under conditions of nutrient limitation [27, 40]. Interestingly, the ability of the plug to activate Pdr1 requires unfolding of bundle, as under normal conditions many of the required hydrophobic residues are sequestered within it [8, 41]. Some of these residues are critical for bundle stability; for example, deletion of the three most C-terminal, YFV, results in bundle instability [26]. Unfolding of the 4HB also results in dissociation of the Zuo1 from the ribosome. Thus, destabilization of the bundle (e.g. perhaps by binding of a small molecule) could initiate a cascade—dissociation from the ribosome and subsequent activation of Pdr1 in the nucleus. Future work will clearly be required to understand the breadth and mechanism of Zuotin’s roles in cellular regulation in both *S*. *cerevisiae* and humans.

## Materials and methods

### Protein expression and purification

DNA encoding residues from 346 to 432 of Mpp11 was amplified by PCR and cloned into a modified pET-28a vector (Novagen, Madison, WI) containing an N-terminal hexahistidine (His6) tag, a thioredoxin (TRX) tag and a cleavable tobacco etch virus (rTEV) protease site. Protein was expressed in *Escherichia coli* Rosetta 2 (DE3) pLysS (Novagen). Isotopic labeling (^15^N and ^15^N,^13^C) of the Mpp11 protein fragment was achieved by growing cells at 37°C in M9 minimal media containing ^15^NH_4_Cl and D-glucose (U-^13^C-99%) (Cambridge Isotope Laboratories, Inc., Andover, MA) and necessary antibiotics. After reaching 0.6 of culture O.D. value, protein expression was induced with a final concentration of 1 mM Isopropyl β-D-1-thiogalactopyranoside (IPTG), and cells were grown for another 6 hours at 30°C. Cells were harvested by centrifugation, resuspended in lysis buffer (50 mM Tris, pH 7.5, 250 mM NaCl, 10 mM imidazole and 5% glycerol) with Roche cOmplete EDTA-free protease-inhibitor cocktail (Millipore-Sigma, St. Louis, MO) and lysed with a French press at 4°C. Initial protein purification was carried out with gravity-flow nickel column chromatography (Thermo Fisher Scientific, Madison, WI). Purification tags were removed by proteolysis with recombinant rTEV protease overnight at 4°C and separated from protein of interest by second gravity-flow nickel chromatography. The Mpp11 fragment was further purified by size-exclusion chromatography using HiLoad 16/60 Superdex 75 column (GE Healthcare, Buckinghamshire, UK) and 20 mM sodium phosphate, pH 7.5, 250 mM NaCl and 5 mM dithiothreitol as buffer conditions. Protein samples were concentrated using Amico Ultra centrifugal filter devices (Merck EMD Millipore Corporation, Billerica, MA) following manufacturer instructions. Protein concentration was determined using Bio-Rad protein assay reagent (BioRad, Hercules, CA) and purity determined by running SDS-PAGE followed by staining with Coomassie Blue. Protein was used immediately or stored at −80°C. All protein NMR samples were used at a concentration of ~300 μM in sodium phosphate buffer, pH 7.5, containing 250 mM NaCl and 5 mM dithiothreitol with 7% ^2^H_2_O and 0.02% NaN_3_.

### NMR data acquisition and processing

Uniformly ^15^N or ^15^N,^13^C labelled samples of Mpp11_346-432_ fragment, with additional Gly and Ser residues at the N-terminus, remaining after cleavage of the expression tag, were used in this NMR study. All NMR data were acquired at NMRFAM (National Magnetic Resonance Facility at Madison) using Varian/Agilent and Bruker spectrometers operating at a variety of magnetic field strengths (600 MHz, 800 MHz and 900 MHz) each equipped with a cryogenic probe. Coordinates are available: PDB ID: 6CGH). All NMR experiments were performed at 5°C, except for the RDC measurements as indicated below. Resonance assignments were obtained from analysis of the following two-dimensional (2D) and three-dimensional (3D) NMR data sets: 2D ^1^H, ^15^N HSQC, 3D HNCACB, 3D CBCA(CO)NH, 2D ^1^H, ^13^C^aliphatic^ HSQC, 3D HBHA(CO)NH, 3D C(CO)NH and 3D H(CCO)NH. Distance restraints for structure determination were obtained from 3D NOESY ^15^N HSQC and 3D NOESY ^13^C^aliphatic/aromatic^ HSQC data. All 3D spectra were recorded using non-uniform sampling (NUS) at a sampling rate of 50%; spectra were reconstructed using the SMILE [42] and NMRPipe [43] software packages. To obtain ^15^N-^1^H residual dipolar coupling (RDC) data, the IPAP-HSQC experiments were carried out on Mpp11 samples aligned by two different media: polyethylene glycol (PEG)/hexanol and bicelles (Avanti Polar Lipids, Alabaster, AL), both doped with cetrimonium bromide (CTAB), at 25°C and 33°C, respectively [44]. The Fourier transform utility of NMRPipe was used to convert the acquired time domain data to the frequency domain [43].

### NMR spectral analysis

NMRFAM-SPARKY was used for NMR spectra analysis [45]. Backbone signals were automatically detected from ^1^H,^15^N HSQC, CBCA(CO)NH and HNCACB spectra by APES [46] and verified manually by combined reference to spectra, peak lists and strip plots [47]. Backbone chemical shifts were assigned by the PINE algorithm [48] and verified by PINE-SPARKY [49]. Side chain chemical shifts were assigned using the predict-and-confirm method with C(CO)NH, HBHA(CO)NH and H(CCO)NH spectra, available in NMRFAM-SPARKY [47]. IPAP-HSQC RDC data were analyzed by NMRPipe [43] and Xplor-NIH [50].

### Structure determination

The solution NMR structure of MPP11 4HB was determined by using the PONDEROSA-C/S software package [51] with the Xplor-NIH calculation option [50]. Resonance assignments, protein sequence, ^15^N-edited NOESY and ^13^C^aliphatic/aromatic^-edited NOESY spectra were used as inputs for the Ponderosa Client program, and the PONDEROSA-X refinement option was set to “submit to Ponderosa Web Server” (http://ponderosa.nmrfam.wisc.edu/ponderosaweb.html). As a result, NOE-derived distance constraints were obtained by the AUDANA algorithm [52], and backbone chemical shift-derived angle constraints were obtained by TALOS-N [53]. Generated constrains were validated by using Ponderosa Analyzer coupled with PyMOL Molecular Graphics System (Schrödinger, LLC) and NMRFAM-SPARKY [45]. After a few iterations of structure calculation using the “Constraints Only-X” option in PONDEROSA-C/S, the final conformers representing the solution structure were determined by using the “implicit water refinement calculation” option, which calculates 100 conformers and selects the 20 best on the basis of the EEFX potential energy term [54]. The agreement between the experimental RDCs measurements and RDC values back-calculated from the structure served to validate the structure. The PSVS software suite was used to further validate the structure [55–58]. In addition, typical NOE signal patterns were found confirming the continuous helical character of the C-terminal region of Helix IV.

### Species phylogeny

Species phylogeny was based on [59, 60] for relative positions of Animalia, Amebozoa, Apicomplexa, Ascomycota and Basidiomycota. The relationships within the major clades were based on: [61, 62] for Animalia; [63–65] for Fungi; [66, 67] for Saccharomycotina,;[68] for Candida; [69] for Saccharomycetaceae,; [70] for Pezizomycotina.

### Sequence alignments, phylogeny, evolution rates and ancestral sequences reconstruction

Protein sequences of the Zuotin family (K09522) were retrieved from the KEGG database v85.1 [71], and aligned using both Clustal Omega v1.2.2 and MAFFT with default parameters [72]. Both multiple alignments were converted into a Hidden Markov Models using hmmbuild program from the HMMER package and forward–backward algorithm was used to compute a posterior probability for each site representing the degree of confidence in each position (residue or gap) of the alignment for each sequence. The quality of both alignments was assessed, by calculating proportion of sites with posterior probability (pp) >0.9, and only the better-supported Clustal Omega alignment was analyzed further. For dN/dS analyses, amino acid positions with pp <0.9 and sequences that introduced >5% gaps were removed from the multiple sequence alignment [73, 74]. The protein alignment was translated to a codon alignment with Pal2Nal using corresponding DNA sequences and trimmed accordingly [75].

To infer protein phylogeny, 1,000 maximum likelihood (ML) searches were performed using RAxML v8.2.10 [76] with 100 rapid bootstrap replicates, under the LG model of amino acid substitution and GAMMA model of rate heterogeneity with four discrete rate categories and the estimate of proportion of invariable sites (LG + I + G) [77], which was determined as the best-fit model by ProtTest v3.2 following Akaike criterion [78]. Domain specific phylogenies were reconstructed based on the fragments of the Zuotin multiple sequence alignment; for J-domain positions 88–157, ZHD-domain positions 175–278, MD-domain positions 279–345 and 4HB-domain positions 346–420 of the human Mpp11 sequence; the extension of human Helix IV and the fungal plug sequence were omitted from this analysis.

Sequence evolution rates for individual domains of Zuotin were calculated based on patristic distances (d) along the branches of the domain trees, calculated under the LG + I + G model of sequence evolution [77] with fixed species topology. The d values were estimated from the common ancestor of animals and fungi to the extant sequences. When the results were compared to those obtained using the topology of the full length Zuotin tree, which places several species at different nodes, no significant differences were observed (S5 and S6 Tables). The distributions of d values were presented as box-and-whisker plots in Matlab R2015a. The differences between the mean values of d were compared with the nonparametric Mann-Whitney U test at significance level of p < 0.01. Ancestral sequences were reconstructed in FastML using empirical Bayes method with Maximum Likelihood reconstruction of insertions and deletions [79].

### dN/dS analyses

Maximum-likelihood analysis of the 4HB dataset was performed with the CODEML program of the PAML software package [80]. A free-ratio branch model (model = 1, NSsites = 0) allowing dN/dS variation along different branches was used to calculate dN/dS ratio estimates for each branch of the 4HB phylogeny. Then, each branch with dN/dS > 1 was specified as a foreground branch and its dN/dS ratio was estimated again using a two-ratio branch model (model = 2, NSsites = 0), allowing positive selection. Both free-ratio and two-ratio branch models were compared with a null one-ratio model (model = 0, NSsites = 0), assuming one dN/dS ratio for all branches of the tree. Likelihood ratio test (LRT) [81] was used to determine which model fits the data better. Branches, which according to both free-ratio and two-ratio branch models had dN/dS > 1, and also passed LRTs are reported as under positive selection.

### Phylogenetic distribution of Pdr1 and Pdr3

The presence of transcription factor Pdr1, and its paralogue Pdr3, was verified using fungi genomic data and a reciprocal best BLAST hit algorithm [82]. Orthology versus paralogy relationships were deduced from Pdr1/3 ML phylogeny, reconstructed with RaxML [76] using the phylogeny reconstruction method described above for the Zuotin phylogeny.

### Hydrophobicity and electrostatic potential calculations

The hydrophobic surfaces of the 4HBs were calculated with the g sas tool from the GROMACS package [83]. The average hydrophobic surfaces buried inside the 4HB were calculated as the difference between the total surface of hydrophobic residues of the selected portions of the 4HB and their surface exposed to the solvent, yielding 5.35 (±0.19) nm^2^ and 3.40 (±0.29) nm^2^ for *S*. *cerevisiae* Zuo1 and human Mpp11, respectively.

The distribution of the electrostatic potential generated by 4HB in an aqueous solution at physiological ionic strength was obtained by integrating the linearized Poisson Boltzmann equation using the Adaptive Poisson–Boltzmann Solver (APBS) software [84]. The concentrations of +1 and -1 ion species were set to 150 mM with an ion exclusion radius of 0.2 nm. Grid size of 120^3^ with grid spacing of 0.069 nm was used. We used the Dirichlet boundary conditions with the boundary potential value determined from a Debye-Huckel model for a single sphere with a point charge, dipole, and quadruple. The electrostatic potential beyond 0.4 nm from a protein surface was visualized using VMD [85].

### *S*. *cerevisiae* rescue tests

As previously described, the *zuo1*::*HIS3* deletion [29] was used. Mpp11 and its variants, which contained a tandem repeat of three HA-tags at the N-terminus, were heterologously expressed from the *GPD1* promoter present from plasmid pRS426. Mutagenesis was carried out using the QuickChange PCR mutagenesis standard protocol (Stratagene, La Jolla, CA).

Yeast cell extracts were prepared for comparison of total protein levels, by growing the cells expressing Mpp11 or its variants at 30°C to an OD_600_ of 0.4–0.5 in selective minimal medium (0.67% yeast nitrogen base without amino acids (US Biological, Marblehead, MA), 2% dextrose), supplemented with required amino acids. For growth assays, equal number of cells were spotted onto selective minimal glucose media containing 250 μg/ml paromomycin. An equivalent of 0.5 OD units of cells were harvested by centrifugation and resuspended in 100 μl of 0.2 M NaOH. After 5 min incubation at room temperature, samples were centrifuged and the resulting pellet resuspended in sodium dodecyl sulfate (SDS) sample buffer and boiled for 5 min. An equal volume of extract was subjected to SDS-PAGE and immunoblot analysis using commercially available antibodies against the HA-tag for detecting Mpp11 (Abcam, Cambridge, MA). Ydj1, a loading control, was detected by previously described polyclonal antibodies against the Ydj1 J-domain [86].

### Miscellaneous

Structures were visualized using PyMOL Molecular Graphics System and VMD [85]. Secondary structure was predicted using the jnet, jhmm and jpssm methods from the Jpred4 web server [87] (http://dx.doi.org/10.1093/nar/gkv332). Results are reported as a confidence score on a 0–9 scale. All chemicals used in this study were purchased from Millipore Sigma unless noted otherwise. Restriction enzymes were purchased from New England BioLabs (Ipswich, MA).

## Supporting information

S1 Fig Organismal phylogeny of 104 eukaryotes from which Zuotin sequences were obtained. The branching pattern was based on a variety of sources as described in Materials and Methods. Green branches represent species harboring transcription factor Pdr1. The presence of the following is indicated by colored symbols: Pdr1, blue dot; Pdr1 paralogue Pdr3, green dot; truncated 4HB, orange triangle; SANT1 and SANT2, gray squares.
(PDF)

S2 Fig Zuotin phylogeny. Maximum Likelihood tree of 104 Zuotin amino acid sequences. Bootstrap support for major clades is indicated.
(PDF)

S3 Fig Phylogeny of Zuotin domains from Saccharomycetaceae (S), Candida (C), Pezizomycotina (P), and Animalia (A), with protists used as an outgroup. Nodes representing common ancestors are indicated by dots: Black—Animalia and Fungi (AncAF), red—Fungi (AncF), cyan—Candida and Saccharomycetaceae clades (AncCS); orange—Saccharomycetaceae (AncS). Numbers above arrows indicate branch length estimated as number of expected amino acid substitutions per site for indicated branches. Species from which Zuotin orthologs were obtained are listed in S1 Fig.
(PDF)

S4 Fig Sequence alignments of the C-terminal fragments of 4HBs from Metazoans and Fungi. (A) Alignment of the C-terminal extension regions of the 4HBs from the indicated Metazoans based on the multiple sequence alignment of all Zuotin sequences from our data set. Residues of more than 50% identity are indicated in black; gaps in the alignment are indicated with dashes. Helical propensity was predicted from the Jpred4 server and are indicated by the red tube below the alignment, together with their confidence score on a 0–9 scale. (B) Alignment of plug sequences from the indicated Fungal species. The presence of the Pdr1, blue dot, and Pdr1 paralogue Pdr3, green dot, in a given species is indicated.
(PDF)

S5 Fig Signatures of positive selection across 4HB domain tree. dN/dS ratios estimated with a free-ratio branch model from CODELM are indicated for each branch of the tree. In cases where dN/dS >1, both the number of nonsynonymous changes and the number of synonymous changes (N:S) are indicated. Statistical support for positive selection is indicated based on the Likelihood Ratio Test (LRT) for two-ratio vs. one-ratio models from CODELM (for details see S2 and S3 Table). Nodes representing common ancestors are indicated by dots: Black–Animalia and Fungi (AncAF), red—Fungi (AncF), cyan–Candida and Saccharomycetaceae clades (AncCS), orange—Saccharomycetaceae (AncS), green—species harboring Pdr1 transcription factor (AncP). Red line marks lineage from AncAF to AncP. Species particularly relevant to this study are in bold.
(PDF)

S6 Fig Foreground branches selected for the two-ratio vs. one ratio model test for positive selection using PAML. Each selected branch (blue) is marked by a number. These numbers correspond to the foreground branches listed in S3 Table. Species particularly relevant to this study are in bold.
(PDF)

S7 Fig Ancestral reconstruction of plug sequences. Inferred ancestral amino acid sequences of plugs are shown for the ancestors indicated by dots: Red—Fungi (AncF), cyan–Candida and Saccharomycetaceae clades (AncCS), orange—Saccharomycetaceae (AncS), green—species harboring Pdr1 transcription factor (AncP). Numbers above each position of the inferred sequences are posterior probabilities (pp) for each ancestral state. Sequence of the plug in *S*. *cerevisiae* (S. cer) is shown for comparison. Large hydrophobic residues are highlighted in yellow. The specific hydrophobic residues that in the *S*. *cerevisiae* plug were demonstrated experimentally to be important for Pdr1 activation are in blue. Conserved Gly residues are highlighted in green.
(PDF)

S1 Table NMR and refinement statistics for 20 conformers of Mpp11 four-helix bundle. (PDF)

S2 Table Likelihood ratio test (LRT) statistics for models of variable selection along branches of the 4HB phylogeny using PAML. (PDF)

S3 Table Likelihood ratio test (LRT) statistics for models of variable selection for specified foreground branches of the 4HB phylogeny using PAML. (PDF)

S4 Table Conservation of plug residues involved in Pdr1 activation in *S*. *cerevisiae*. (PDF)

S5 Table Evolution rates (substitutions/position) estimated based on the topology of the species tree (see S1 Fig). (PDF)

S6 Table Evolution rates (substitutions/position) estimated based on the topology of the Zuotin tree (see S2 Fig). (PDF)
