# Supplementary figures and images for: Structure and evolution of the 4-helix bundle domain of Zuotin, a J-domain protein co-chaperone of Hsp70
Source: PLoS One. 2019 May 15;14(5):e0217098. doi: 10.1371/journal.pone.0217098 (PMC6519820; doi:10.1371/journal.pone.0217098)

S1 Fig

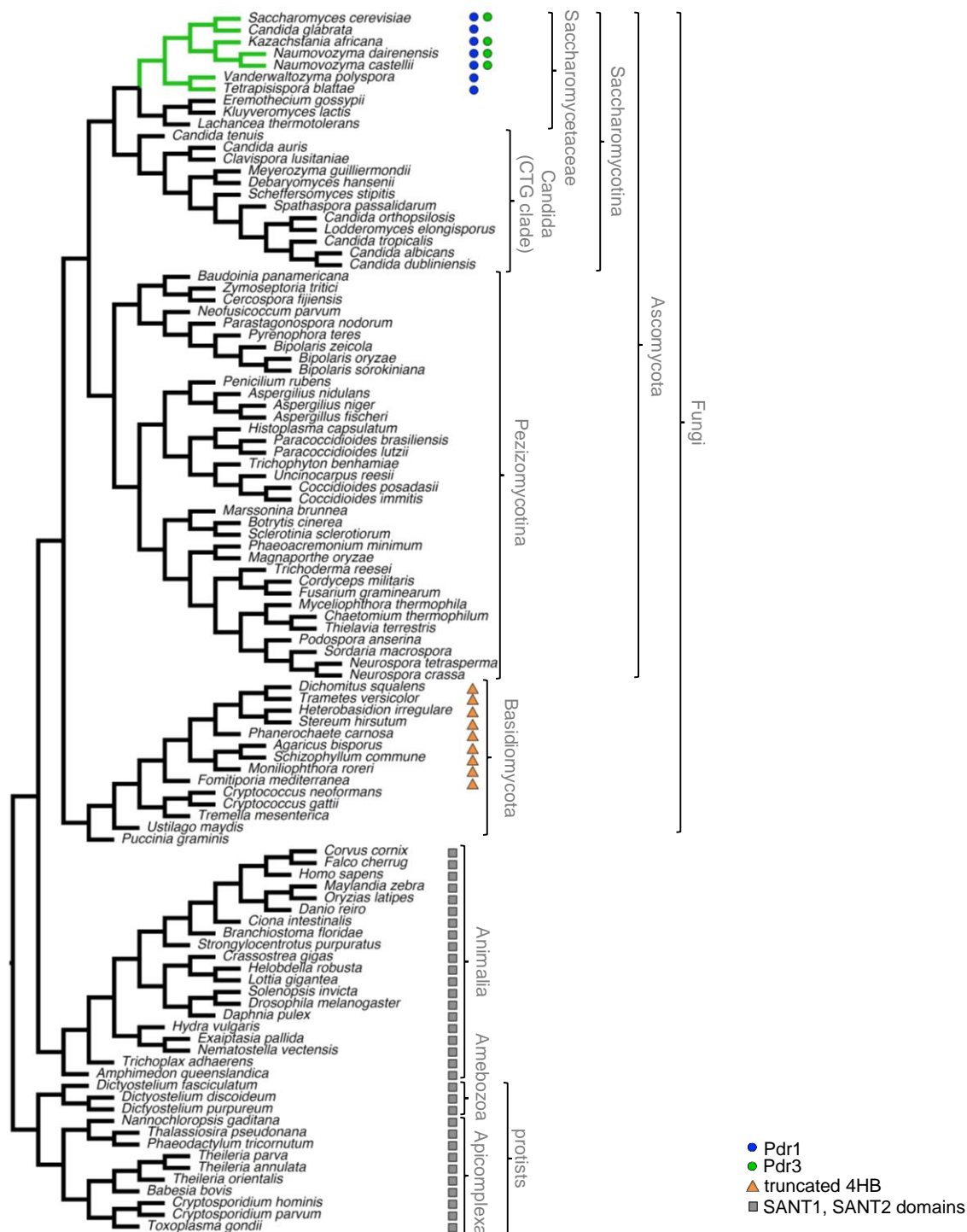

Supplement: S1 Fig — The branching pattern was based on a variety of sources as described in Materials and Methods. Green branches represent species harboring transcription factor Pdr1. The presence of the following is indicated by colored symbols: Pdr1, blue dot; Pdr1 paralogue Pdr3, green dot; truncated 4HB, orange triangle; SANT1 and SANT2, gray squares. (PDF) [file pone.0217098.s001.pdf]

S2 Fig

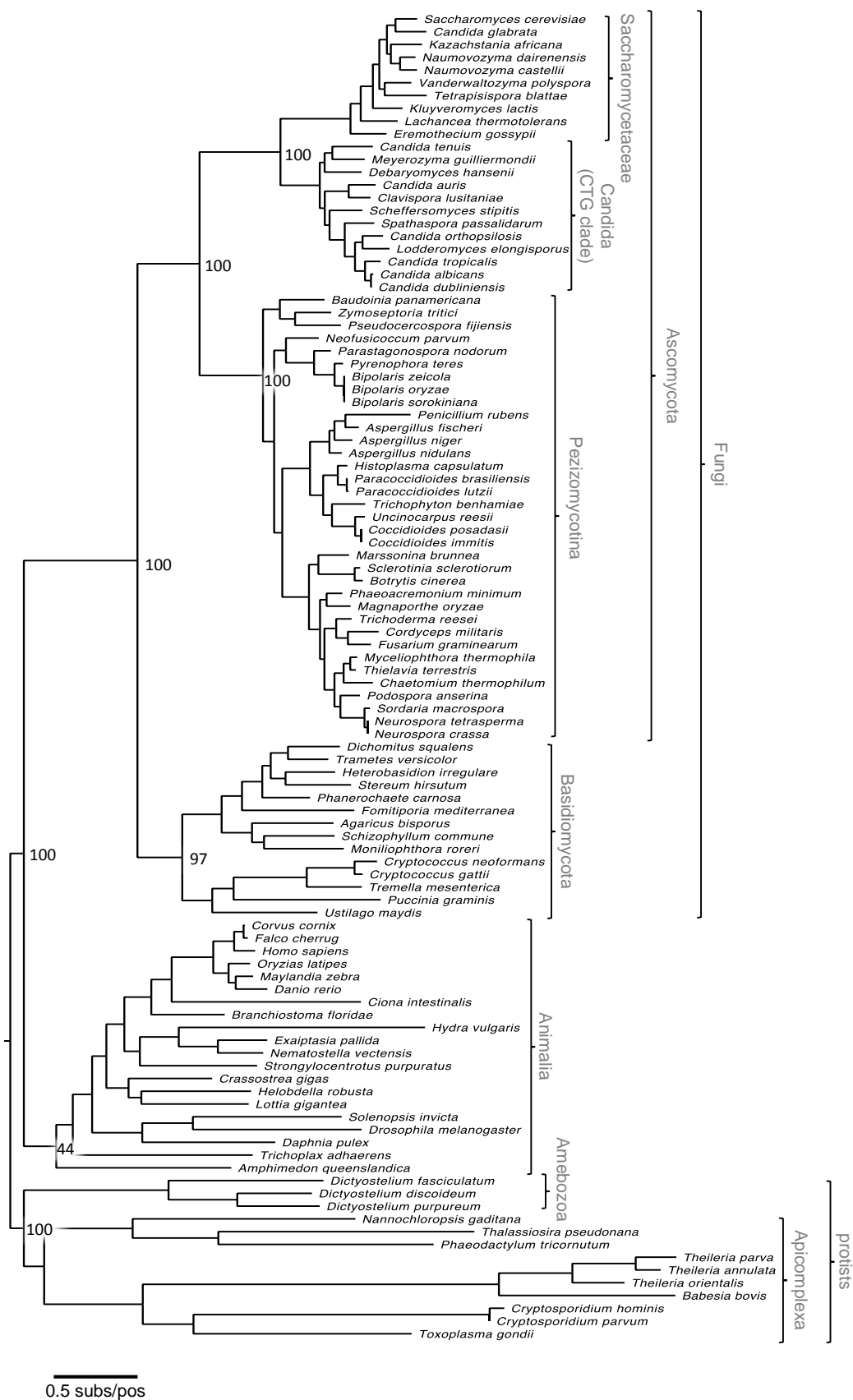

Supplement: S2 Fig — Maximum Likelihood tree of 104 Zuotin amino acid sequences. Bootstrap support for major clades is indicated. (PDF) [file pone.0217098.s002.pdf]

S3 Fig

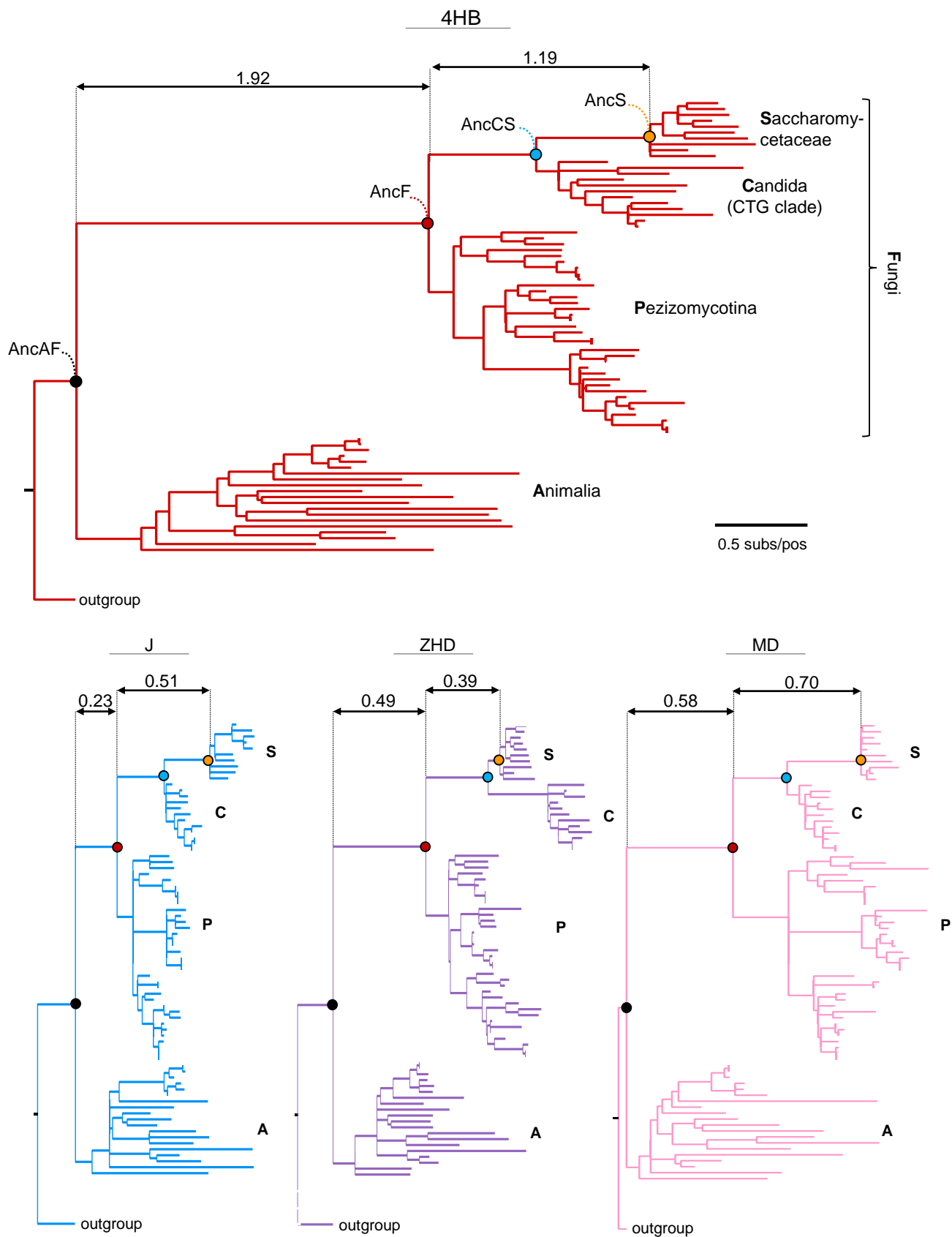

Supplement: S3 Fig — Nodes representing common ancestors are indicated by dots: Black—Animalia and Fungi (AncAF), red—Fungi (AncF), cyan—Candida and Saccharomycetaceae clades (AncCS); orange—Saccharomycetaceae (AncS). Numbers above arrows indicate branch length estimated as number of expected amino acid substitutions per site for indicated branches. Species from which Zuotin orthologs were obtained are listed in S1 Fig. (PDF) [file pone.0217098.s003.pdf]

S5 Fig

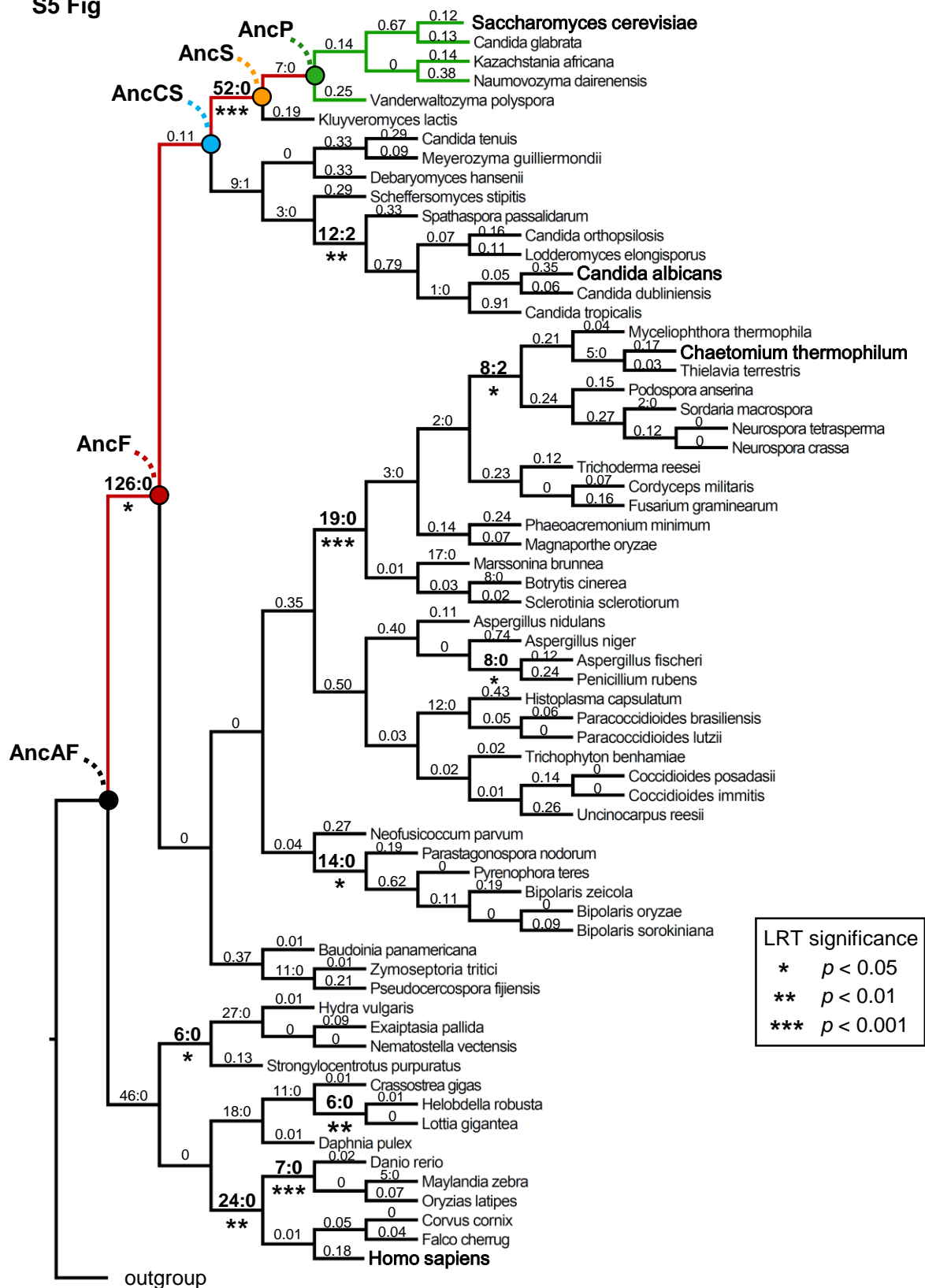

Supplement: S5 Fig — dN/dS ratios estimated with a free-ratio branch model from CODELM are indicated for each branch of the tree. In cases where dN/dS >1, both the number of nonsynonymous changes and the number of synonymous changes (N:S) are indicated. Statistical support for positive selection is indicated based on the Likelihood Ratio Test (LRT) for two-ratio vs. one-ratio models from CODELM (for details see S2 and S3 Table). Nodes representing common ancestors are indicated by dots: Black–Animalia and Fungi (AncAF), red—Fungi (AncF), cyan–Candida and Saccharomycetaceae clades (AncCS), orange—Saccharomycetaceae (AncS), green—species harboring Pdr1 transcription factor (AncP). Red line marks lineage from AncAF to AncP. Species particularly relevant to this study are in bold. (PDF) [file pone.0217098.s005.pdf]

S6 Fig

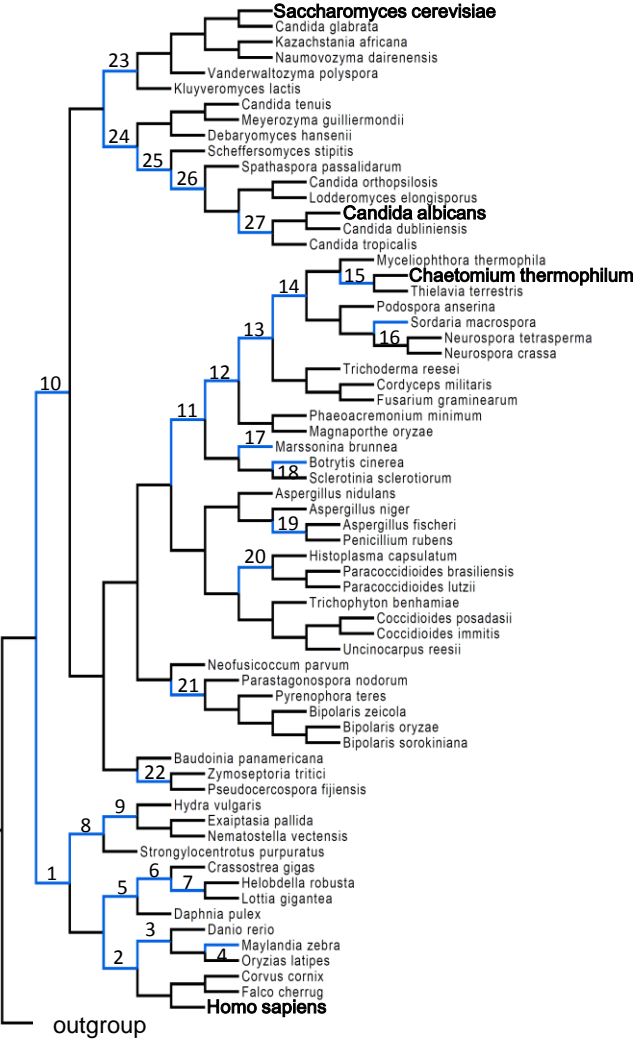

Supplement: S6 Fig — Each selected branch (blue) is marked by a number. These numbers correspond to the foreground branches listed in S3 Table. Species particularly relevant to this study are in bold. (PDF) [file pone.0217098.s006.pdf]

**S7 Fig**

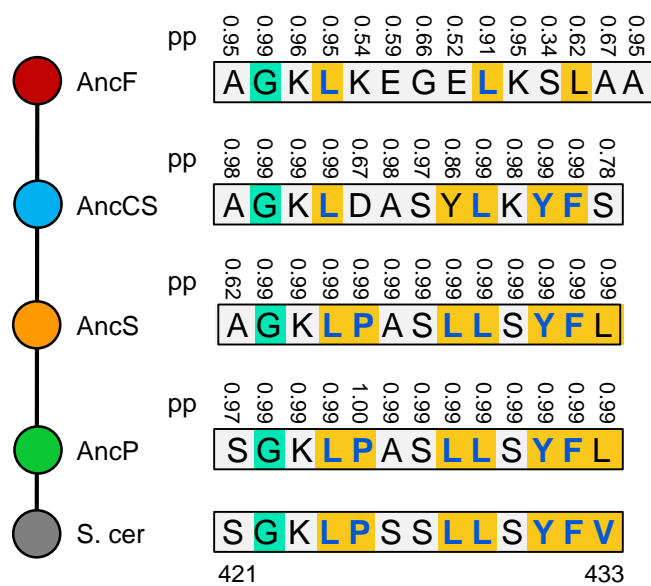

Supplement: S7 Fig — Inferred ancestral amino acid sequences of plugs are shown for the ancestors indicated by dots: Red—Fungi (AncF), cyan–Candida and Saccharomycetaceae clades (AncCS), orange—Saccharomycetaceae (AncS), green—species harboring Pdr1 transcription factor (AncP). Numbers above each position of the inferred sequences are posterior probabilities (pp) for each ancestral state. Sequence of the plug in S. cerevisiae (S. cer) is shown for comparison. Large hydrophobic residues are highlighted in yellow. The specific hydrophobic residues that in the S. cerevisiae plug were demonstrated experimentally to be important for Pdr1 activation are in blue. Conserved Gly residues are highlighted in green. (PDF) [file pone.0217098.s007.pdf]
