# Supplementary material for: Structure and evolution of the 4-helix bundle domain of Zuotin, a J-domain protein co-chaperone of Hsp70
Source: PLoS One. 2019 May 15;14(5):e0217098. doi: 10.1371/journal.pone.0217098 (PMC6519820; doi:10.1371/journal.pone.0217098)
Supplement: S4 Fig — (A) Alignment of the C-terminal extension regions of the 4HBs from the indicated Metazoans based on the multiple sequence alignment of all Zuotin sequences from our data set. Residues of more than 50% identity are indicated in black; gaps in the alignment are indicated with dashes. Helical propensity was predicted from the Jpred4 server and are indicated by the red tube below the alignment, together with their confidence score on a 0–9 scale. (B) Alignment of plug sequences from the indicated Fungal species. The presence of the Pdr1, blue dot, and Pdr1 paralogue Pdr3, green dot, in a given species is indicated. (PDF) [file pone.0217098.s004.pdf]

S4 Fig

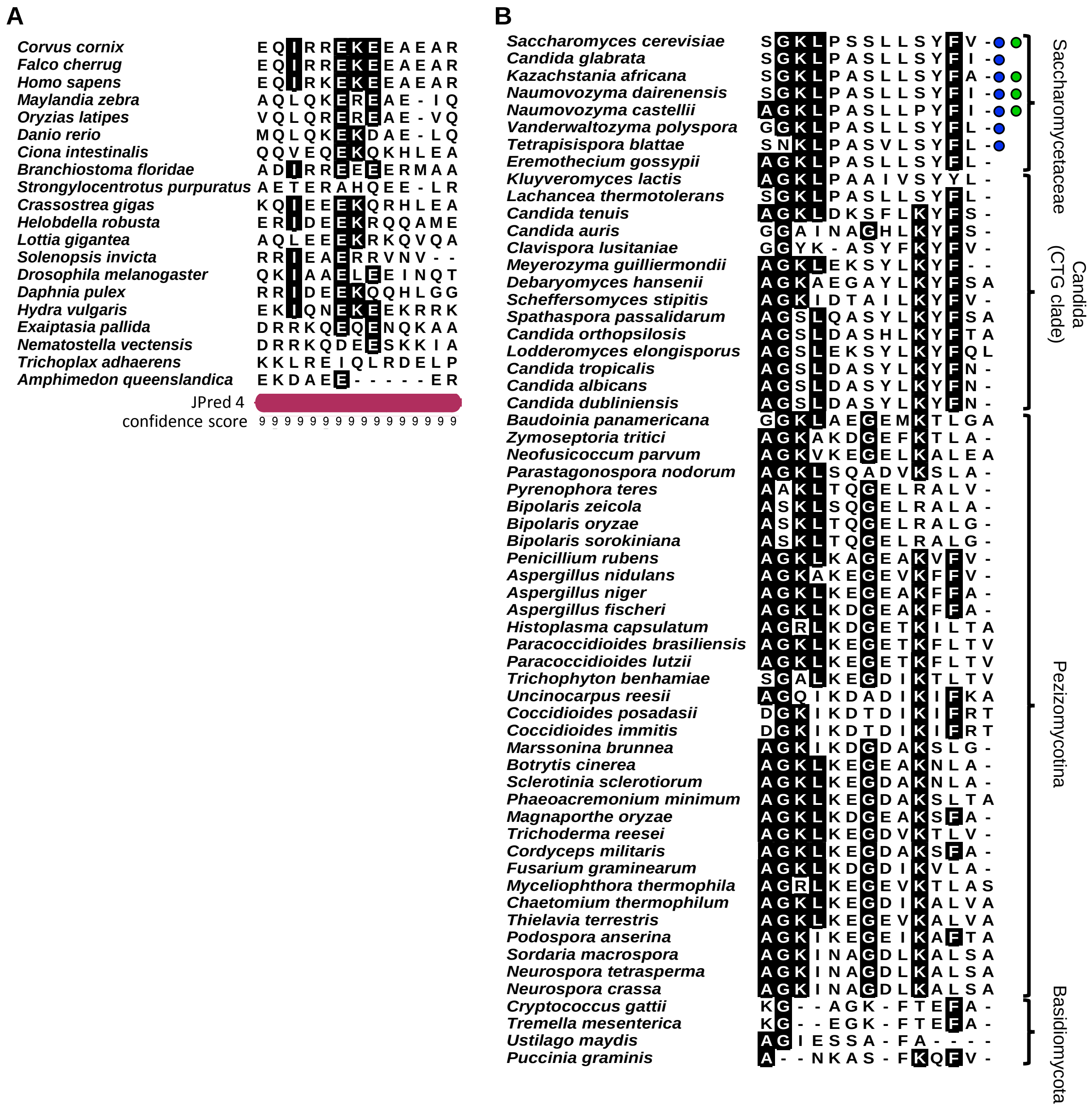
A
Corvus cornix EQIRREKEEAEAR
Falco cherrug EQIRREKEEAEAR
Homo sapens EQIRKEKEEAEAR
Maylandia zebra AQLQKEREAE-IQ
Oryzias latipes VQLQREREAE-VQ
Danio rerio MQLQKEKDAE-LQ
Ciona intestinalis QQVEQEKQKHLEA
Branchiostoma floridae ADIRREEEERMAA
Strongylocentrotus purpuratus AETERAHQEE-LR
Crassostrea gigas KQIEEEKQRHLEA
Helobdella robusta ERIDEEKRQQAME
Lottia gigantea AQLEEEKRKQVQA
Solenopsis invicta RRIEAERRVNV--
Drosophila melanogaster QKIAAELEEINQT
Daphnia pulex RRIDEEKQQHLGG
Hydra vulgaris EKIQNEKEEKRRK
Exaiptasia pallida DRRKQEQENQKAA
Nematostella vectensis DRRKQDEESKKIA
Trichoplax adhaerens KKLREIQLRDELP
Amphimedon queenslandica EKDAEE-----ER
JPred 4
confidence score 9999999999999999
B
Saccharomyces cerevisiae SGKLPSSLLSYFV-
Candida glabrata SGKLPASLLSYFI-
Kazachstania africana SGKLPASLLSYFA-
Naumovozyma dairenensis SGKLPASLLSYFI-
Naumovozyma castellii AGKLPASLLPYFI-
Vanderwaltozyma polyspora GGKLPASLLSYFL-
Tetrapisispora blattae SNKLPASVLSYFL-
Eremothecium gossypii AGKLPASLLSYFL-
Kluyveromyces lactis AGKLPAAIVSYYL-
Lachancea thermotolerans SGKLPASLLSYFL-
Candida tenuis AGKLDKSFLKYFS-
Candida auris GGAINAGHLKYFS-
Clavispora lusitaniae GGYK-ASYFKYFV-
Meyerozyma guilliermondii AGKLEKSYLKYF--
Debaryomyces hansenii AGKAEGAYLKYFSA
Scheffersomyces stipitis AGKIDTAILKYFV-
Spathaspora passalidarum AGSLQASYLKYFSA
Candida orthopsilosis AGSLDASHLKYFTA
Lodderomyces elongisporus AGSLEKSYLKYFQL
Candida tropicalis AGSLDASYLKYFN-
Candida albicans AGSLDASYLKYFN-
Candida dubliniensis AGSLDASYLKYFN-
Baudoinia panamericana GGKLAEGEMKTLGA
Zymoseptoria tritici AGKAKDGEFKTLA-
Neofusicoccum parvum AGKVKEGELKALEA
Parastagonospora nodorum AGKLSQADVKSLA-
Pyrenophora teres AAKLTQGELRALV-
Bipolaris zeicola ASKLSQGELRALA-
Bipolaris oryzae ASKLTQGELRALG-
Bipolaris sorokiniana ASKLTQGELRALG-
Penicillium rubens AGKLKAGEAKVFV-
Aspergillus nidulans AGKAKEGEVKFFV-
Aspergillus niger AGKLKEGEAKFFA-
Aspergillus fischeri AGKLKDGEAKFFA-
Histoplasma capsulatum AGRLKDGETKILTA
Paracoccidioides brasiliensis AGKLKEGETKFLTV
Paracoccidioides lutzii AGKLKEGETKFLTV
Trichophyton benhamiae SGALKEGDIKTLTV
Uncinocarpus reesii AGQIKDADIKIFKA
Coccidioides posadasii DGKIKDTDIKIFRT
Coccidioides immitis DGKIKDTDIKIFRT
Marssonina brunnea AGKIKDGDAKSLG-
Botrytis cinerea AGKLKEGEAKNLA-
Sclerotinia sclerotiorum AGKLKEGDAKNLA-
Phaeoacremonium minimum AGKLKEGDAKSLTA
Magnaporthe oryzae AGKLKDGEAKSFA-
Trichoderma reesei AGKLKEGDVKTLV-
Cordyceps militaris AGKLKEGDAKSFA-
Fusarium graminearum AGKLKDGDIKVLA-
Myceliophthora thermophila AGRLKEGEVKTLAS
Chaetomium thermophilum AGKLKEGDIKALVA
Thielavia terrestris AGKLKEGEVKALVA
Podospora anserina AGKIKEGEIKAFTA
Sordaria macrospora AGKINAGDLKALSA
Neurospora tetrasperma AGKINAGDLKALSA
Neurospora crassa AGKINAGDLKALSA
Cryptococcus gattii KG--AGK-FTEFA-
Tremella mesenterica KG--EGK-FTEFA-
Ustilago maydis AGIESSA-FA----
Puccinia graminis A--NKAS-FKQFV-
Saccharomycetaceae
Candida (CTG clade)
Pezizomycotina
Basidiomycota
