# Supplementary material for: Structure and evolution of the 4-helix bundle domain of Zuotin, a J-domain protein co-chaperone of Hsp70
Source: PLoS One. 2019 May 15;14(5):e0217098. doi: 10.1371/journal.pone.0217098 (PMC6519820; doi:10.1371/journal.pone.0217098)
Supplement: S1 Table — (PDF) [file pone.0217098.s008.pdf]

S1 Table NMR and refinement statistics for 20 conformers of Mpp11 four-helix bundle.

**NOE-derived distance constraints**	
Short range [$\|i-j\|\leq 1$]	754
Medium range [$1<\|i-j\|\leq 5$]	337
Long range [$\|i-j\|>5$]	158
Total	1249
**TALOS-N-derived dihedral angle constraints**	
ϕ	80
ψ	80
Total	160
**NOE pattern-derived Hydrogen-bond constraints**	120
**Residual dipolar coupling (RDC) constraints**	
using PEG/hexanol as aligning media	74
using bicelles as aligning media	63
**Average atomic RMSD to the mean PONDEROSA coordinates [Å]**	
Backbone heavy atoms N, C^{α}, CO (residues 348-430)	0.28
All heavy atoms (residues 348-430)	0.89
**PROCHECK raw-score/Z-score (ϕ-ψ)**	0.59 / 2.64
**Ramachandran plot summary from PROCHECK [%]**	
Most favored regions	95.2
Additionally allowed regions	4.8
Generously allowed regions	0
Disallowed regions	0
**MOLPROBITY mean score/clash score**	13.91 / -0.86
**Ramachandran plot summary from MOLPROBITY [%]**	
Most favored regions	98.1
Allowed regions	1.9
Disallowed regions	0
**PONDEROSA pseudo-potential energy [kJ/mol]**	3419.47
**Consistent violations**	
Distance	0
Dihedral angle	0
Van der Waals	0
