# Supplementary material for: Structure and evolution of the 4-helix bundle domain of Zuotin, a J-domain protein co-chaperone of Hsp70
Source: PLoS One. 2019 May 15;14(5):e0217098. doi: 10.1371/journal.pone.0217098 (PMC6519820; doi:10.1371/journal.pone.0217098)
Supplement: S2 Table — (PDF) [file pone.0217098.s009.pdf]

S2 Table Likelihood ratio test (LRT) statistics for models of variable selection along branches of the 4HB phylogeny using PAML.

Model	lnL*	k**	free-ratio vs one-ratio	
			2ΔlnL***	*p*-value
one-ratio model<br>same dN/dS for all branches	-11195.73	-	-	-
free-ratio model<br>different dN/dS ratio for different branches	-10998.02	128	395.42	<0.001

* likelihood values are provided in the natural log (probability of observing the data given the parameter values).
** degrees of freedom for the LRT, equal to one less than the total number of branches in the tree.
*** 2ΔlnL – twice log likelihood difference.
