# Supplementary material for: Structure and evolution of the 4-helix bundle domain of Zuotin, a J-domain protein co-chaperone of Hsp70
Source: PLoS One. 2019 May 15;14(5):e0217098. doi: 10.1371/journal.pone.0217098 (PMC6519820; doi:10.1371/journal.pone.0217098)
Supplement: S3 Table — (PDF) [file pone.0217098.s010.pdf]

S3 Table Likelihood ratio test (LRT) statistics for models of variable selection for specified foreground branches of the 4HB phylogeny using PAML.

Model	foreground branch	lnL*	k**	dN/dS	two-ratio vs one-ratio	
					2ΔlnL***	*p*-value
one-ratio model same dN/dS for all branches	-	-11195.73	-	0.12596	-	-
two-ratio model different dN/dS for specified foreground branch	1	-11194.23	1	0.00411	3.003642	-
	2	-11192.32	1	0.00565	6.82368	<0.01
	3	-11188.94	1	0.00425	13.58204	<0.001
	4	-11195.64	1	0.08985	0.186084	-
	5	-11194.87	1	0.00889	1.73491	-
	6	-11195.65	1	0.01470	0.17596	-
	7	-11191.50	1	0.00017	8.46082	<0.01
	8	-11193.73	1	0.00066	4.003422	<0.05
	9	-11194.29	1	0.00198	2.888888	-
	10	-11193.09	1	0.00896	5.279972	<0.05
	11	-11186.71	1	999.00000	18.050736	<0.001
	12	-11195.68	1	0.19311	0.10305	-
	13	-11195.11	1	999.00000	1.241894	-
	14	-11193.61	1	5.53995	4.248096	<0.05
	15	-11194.99	1	999.00000	1.476642	-
	16	-11194.05	1	999.00000	3.3642	-
	17	-11195.49	1	0.18203	0.496082	-
	18	-11194.95	1	0.34710	1.56347	-
	19	-11193.09	1	999.00000	5.297992	<0.05
	20	-11195.39	1	0.06497	0.692688	-
	21	-11192.59	1	999.00000	6.29297	<0.05
	22	-11194.92	1	999.00000	1.633222	-
	23	-11187.23	1	999.00000	17.010218	<0.001
	24	-11195.61	1	0.04210	0.246366	-
	25	-11194.16	1	999.00000	3.159068	-
	26	-11190.96	1	999.00000	9.554454	<0.01
	27	-11195.66	1	999.00000	0.15786	-

* likelihood values are provided in the natural log (probability of observing the data given the parameter values)
** degrees of freedom for the LRT
*** 2ΔlnL – twice log likelihood difference
