# Supplementary material for: Structure and evolution of the 4-helix bundle domain of Zuotin, a J-domain protein co-chaperone of Hsp70
Source: PLoS One. 2019 May 15;14(5):e0217098. doi: 10.1371/journal.pone.0217098 (PMC6519820; doi:10.1371/journal.pone.0217098)
Supplement: S4 Table — (PDF) [file pone.0217098.s011.pdf]

**S4 Table** Conservation of plug residues involved in Pdr1 activation in *S. cerevisiae*

| **Residues of the plug important for Pdr1 activation*** | **Conservation in Pdr1 clade (7 sequences)** | **Conservation in Saccharomycetaceae without Pdr1 (3 sequences)** |
| --- | --- | --- |
| L424 | 7L | 3L |
| P425 | 7P | 3P |
| L428 | 6L, 1V | 2L, 1I |
| L429 | 7L | 2L, 1V |
| Y431 | 7Y | 3Y |
| F432 | 7F | 2F, 1Y |
| V433 | 3I, 2L, 1A, 1V | 3 L |

* Amino acid positions shown are for *S. cerevisiae* 4HB
