# Supplementary material for: Structure and evolution of the 4-helix bundle domain of Zuotin, a J-domain protein co-chaperone of Hsp70
Source: PLoS One. 2019 May 15;14(5):e0217098. doi: 10.1371/journal.pone.0217098 (PMC6519820; doi:10.1371/journal.pone.0217098)
Supplement: S6 Table — (PDF) [file pone.0217098.s013.pdf]

S6 Table Evolution rates (substitutions/position) estimated based on the topology of the Zuotin tree (see S2 Fig.).

domain* / clade	J		ZHD		MD		4HB	
	avg	SD	avg	SD	avg	SD	avg	SD
Saccharomycetaceae	1.11	0.07	1.35	0.06	1.59	0.08	3.74	0.12
Candida	0.82	0.08	1.52	0.03	1.35	0.03	3.34	0.22
Pezizomycotina	0.77	0.12	1.08	0.22	1.62	0.18	2.95	0.15
Animalia	0.86	0.06	0.79	0.05	0.80	0.06	2.32	0.38

* J-domain (J), ZHD-domain (ZHD), MD-domain (MD) 4HB-domain (4HB)
